# Risks and outcomes of extubation failure after acute stanford Type A aortic dissection: a retrospective cohort study based on propensity score matching

**DOI:** 10.1186/s13019-026-04454-6

**Published:** 2026-07-12

**Authors:** Luo zhuo, Li Li, Huan Xu, Lei Zou, Yinying Xue, Xiaochun Song, Cui Zhang

**Affiliations:** Department of Critical Care Medicine, Nanjing First Hospital, Nanjing Medical University, 68 Changle Road, Nanjing, 210006 Jiangsu Province China

**Keywords:** Acute Stanford Type A Aortic Dissection, Propensity Score Matching, Extubation Failure, Risk Prediction

## Abstract

**Background:**

Extubation failure is a serious adverse event after cardiac surgery, particularly following acute Stanford type A aortic dissection (ATAAD) repair, with rates exceeding those after routine general anesthesia. This study aimed to identify risk factors and compare outcomes between patients who experienced extubation successfully and those who experienced extubation failure after ATAAD surgery.

**Methods:**

We included consecutive patients admitted to the ICU following ATAAD surgery between January 2016 and December 2021. Using 1:1 propensity score matching (PSM) on the basis of patient characteristics and medical history, we compared matched cohorts with successful extubation and those with failed extubation. Receiver operating characteristic (ROC) curve analysis was used to identify optimal predictive thresholds. Both univariate and multivariable logistic regression analyses were performed to identify independent predictors of extubation failure. We analysed the reasons for extubation failure.

**Results:**

After PSM (18 patients per group), patients with extubation failure had higher preoperative APACHE II scores (12.89 ± 3.07 vs. 10.00 ± 1.94; *P* = 0.002) and GERAADA scores (25.14 ± 6.25 vs. 19.06 ± 10.81; *P* = 0.046). ROC analysis identified thresholds: APACHE II > 12.5 (AUC 0.744) and GERAADA > 21.9% (AUC 0.832); DeLong test *P* = 0.485. Multivariable analysis confirmed APACHE II as an independent predictor (OR = 1.66, *P* = 0.023). Among extubation failure patients, early reintubation (≤ 24 h) occurred in 5 (23.8%, primarily surgical complications) and late reintubation (> 24 h) in 16 (76.2%, predominantly pulmonary infection/secretion retention). No significant differences were observed in preoperative/peri-extubation oxygenation, carbon dioxide retention, ICU stay, or mortality rates between groups.

**Conclusions:**

Preoperative APACHE II score > 12.5 and GERAADA score > 21.9% may identify patients at increased risk of extubation failure after ATAAD surgery. Given that delayed failure was primarily driven by impaired airway clearance, post-extubation management should prioritize airway clearance strategies alongside conventional oxygen support. These exploratory findings require prospective validation.

## Introduction

Extubation failure after aortic dissection surgery is a high-risk clinical event associated with increased disease severity, extended hospital stays, and adverse outcomes. The incidence of extubation failure in patients who are scheduled for extubation after surgical procedures ranges from 0.00014% to 0.00172% [1]. However, this probability increases to 1.06% in patients following cardiac surgery and 2.2% among those undergoing vascular surgery [2–3]. For patients specifically undergoing aortic dissection surgery, the probability is even greater, reaching 7.5% in a small-sample, single-center study conducted in China [4]. Risk factors for extubation failure in patients following surgery for acute aortic dissection primarily include advanced age, obesity, male, emergency surgery, surgical complexity, prolonged cardiopulmonary bypass (CPB) time, excessive postoperative drainage, pericardial tamponade, dissection-related lung injury, postoperative stroke or hemiplegia, and other related complications. The need for reintubation or tracheostomy in patients who fail extubation results in a longer intensive care unit (ICU) length of stay (LOS), higher in-hospital mortality, and poorer long-term clinical outcomes [5–6]. This leads to hesitation among ICU physicians with a critical care background when making extubation decisions, as they typically conduct repeated assessments before extubation to ensure optimal airway clearance capacity and oxygenation status. However, this approach contradicts the current concepts of early extubation, such as the Fast Track Cardiac Anaesthesia (FTCA) and the Enhanced Recovery After Surgery (ERAS), which advocate timely extubation [7–8].

Given the aforementioned context, this project employs the PSM method to construct baseline-matched cohort data of patients who underwent surgery for acute type A aortic dissection (ATAAD) with successful extubation and those with failed extubation from observational cohort data. We aimed to identify differences in risk factors and outcomes between the two groups through this approach.

## Methods

### Study design and patients

**Fig. 1:**
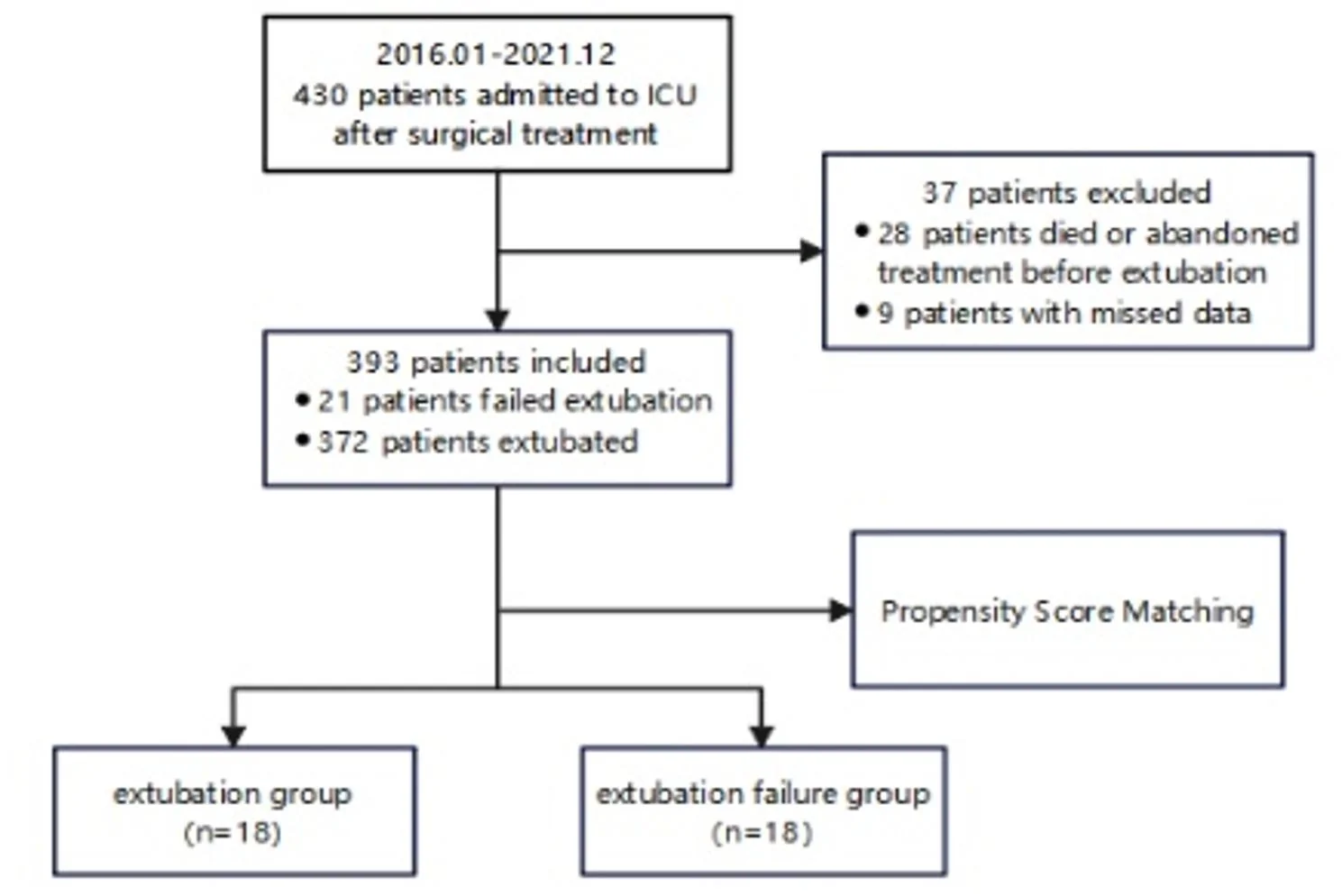
Flow chat

This single-center, retrospective observational study was performed in the ICU at Nanjing First Hospital from January 2016 to December 2021. This study included patients who underwent surgery for ATAAD during this period (including those who underwent aortic surgery only as well as those who had concurrent coronary or valvular procedures) while excluding patients under 18 years of age and those with an expected survival of less than 24 h. Among the 430 patients initially considered, 393 (372 successful extubations and 21 failed extubations) were ultimately included in the data analysis after excluding those with missing data or who died or abandoned treatment prior to extubation. We performed 1:1 PSM via R (version 4.4.2) on the basis of preoperative factors such as age, BMI, smoking history, alcohol consumption history, history of structural lung disease and lung surgery, history of stroke, and history of cardiac surgery (Fig. 1). This process ultimately yielded the cohort required for analysis.

### Peri-extubation patient management protocol

As this is a retrospective study, the extubation procedure for included patients could not be intervened during data collection. All patients were managed according to the standard extubation protocol of our center. Upon admission to the ICU post-surgery, if patients did not present with severe agitation or significant patient-ventilator asynchrony, sequential sedation was not routinely administered. Medication and ventilator settings were adjusted solely based on the patient’s clinical condition to maintain stable oxygenation, hemodynamics, and homeostasis of internal environment and electrolyte balance.

Specifically, during the emergence from anesthesia, patients undergo comprehensive physiological optimization. This includes adjusting fluid status, optimizing vasoactive medications, providing organ function support, and, when necessary, administering blood transfusions, performing airway clearance, and implementing lung recruitment maneuvers. Awakening without sedation was permitted once the following criteria were satisfied: ventilator settings with FiO₂ ≤60% and PEEP ≤ 10 cmH₂O; hemodynamic stability, defined as norepinephrine ≤ 0.1 µg/kg/min, dopamine ≤ 8 µg/kg/min, dobutamine ≤ 8 µg/kg/min, and epinephrine ≤ 0.06 µg/kg/min, with neither use of terlipressin nor posterior pituitary extract; and absence of any clinical condition necessitating continued mechanical ventilation, such as potential reoperation, new-onset myocardial or cerebral infarction, frequent ventricular arrhythmias, aortic dissection–related lung injury and so on. Patients not fulfilling these criteria received short-acting sedation until all specified conditions were satisfied. All patients underwent pain assessment (critical care pain observation tool for sedated or anesthetized patients, Numerical Rating Scale for awake patients), and were given corresponding analgesic treatment.

Once patients were conscious and had regained limb muscle strength of grade 4 or above, they proceeded to the Spontaneous Breathing Trial (SBT) conducted in Pressure Support Ventilation (PSV) mode. Ventilator settings were adjusted to FiO₂ ≤ 40%, PEEP 6–8mmHg, and Pressure Support (PS) 8–10cmH₂O. The patient was observed for over 30 min. If vital signs (HR, RR, BP, SpO₂) remained stable without significant fluctuations, and subsequent arterial blood gas analysis showed a P/F ratio ≥ 150 with no hypoxia-related increase in lactate levels, the endotracheal tube was extubated. For patients with mechanical ventilation duration exceeding 24 h, a cuff leak test was added prior to extubation.

All bedside rehabilitation activities at our center are scheduled during daytime hours. For patients extubated in the afternoon or at night, planned mobilization commences the following day. Patients extubated in the morning are incorporated into the activity plan on the same afternoon. Based on the patient’s general condition, activities are selected from among bed-based, bedside, and ward-level exercises. The daily activity plan is jointly assessed during ward rounds by the attending physician, respiratory therapist, and primary nurse. Ultra-early rehabilitation activities including leg-dangling sitting (on bed), or assisted standing and transferring to sit in a bedside chair—facilitate diaphragmatic excursion, augment thoracic capacity, and decrease gravity-dependent pulmonary atelectasis via alterations in body position. Sustained rehabilitation over the medium to long term like ward ambulation (some of them walking within the ward while still connected to the ventilator) helps critically ill patients attenuate ICU-acquired weakness and maintain strength in both peripheral and core muscle groups.

### Data collection

Owing to the extended study duration, data were collected via both paper-based records and electronic charting systems. The following variables were extracted: preoperative and postoperative severity scores: Acute Physiology and Chronic Health Evaluation Ⅱ (APACHE Ⅱ), the German Registry for Acute Aortic Dissection Type A (GERAADA) and the European System for Cardiac Operative Risk Evaluation (EuroScore). Postoperative complications: Stroke, paraplegia, hemorrhage, and pulmonary infection. Surgical parameters: Operation time, CPB time, and aortic cross-clamp (ACC) time. Blood gas analysis: Partial pressure of arterial carbon dioxide (PaCO_2_) and the ratio of PaCO_2_ to fractional inspired oxygen (PaO_2_/FiO_2_) were measured preoperatively and during peri-extubation. Outcome measures: Duration of mechanical ventilation, ICU LOS, hospital LOS, in-hospital mortality, and 90-day mortality.

### Statistical analysis

Basic statistical analyses were performed via SPSS 19.0 (IBM, Armonk, NY, USA). Continuous variables are expressed as the mean ± standard deviation (SD) or median (interquartile range [IQR]) and were compared via Student’s *t* test, Welch’s *t* test, or the Mann‒Whitney *U* test, depending on the data distribution and variance homogeneity. Categorical variables are reported as proportions and were analysed with the chi-square test or Fisher’s exact test, as appropriate. ROC curve analysis was performed using R software (version 4.4.2), and the DeLong test was used to compare the area under the curve (AUC) between APACHE II and GERAADA scores. The ROC curves were generated using GraphPad Prism (version 9.1.0) for visualization. To further eliminate the remaining confounding factors, we conducted a univariate logistic regression analysis using R to identify the factors associated with failed extubation. For continuous variables, odds ratios (OR) with 95% confidence intervals (CI) were calculated. For categorical variables, Fisher’s exact test was used due to the small sample size.

Variables with *P* < 0.10 in univariate analysis were considered for inclusion in the multivariable model. Given the limited sample size (*n* = 36, with 18 events), we constructed a parsimonious multivariable logistic regression model including only the two strongest predictors identified in univariate analysis (preoperative APACHE Ⅱ and GERAADA scores). This approach respects the rule of thumb requiring 10–20 events per predictor variable. Multicollinearity was evaluated using variance inflation factors (VIF). A VIF value > 5 was considered indicative of problematic multicollinearity.

A two-tailed *P* value < 0.05 was considered statistically significant. Given the exploratory nature of this analysis with small sample size, we also considered variables with *P* < 0.10 as showing a trend toward significance in univariate analysis.

## Results

### Demographic and clinical characteristics

This study included a total of 393 patients with a mean age of 53.39 ± 11.27 years. Among them, 302 were male (76.8% of the total), 282 (71.76%) were overweight body mass index [BMI] ≥ 24), 160 (40.71%) had a history of smoking, and 98 (24.94%) had a history of alcohol consumption. Additionally, 16 patients had pulmonary organic diseases or had undergone partial lung resection surgery for various reasons, 25 had a history of stroke, and 4 had undergone cardiac surgery due to valvular heart disease or coronary atherosclerotic heart disease. Before PSM, significant differences were observed between the successful extubation and failed extubation groups in terms of sex distribution, BMI, and history of stroke (*P* < 0.05). After 1:1 PSM, no statistically significant differences were observed between the two groups, indicating balanced baseline characteristics. Baseline demographic and clinical characteristics are summarized in Table 1.

**Table 1:** Characteristics of patients

**Variables**		Before PSM			After PSM			
		Extubation group	failure group	*P*	Extubation group	Failure group	*P*	SMD
		*n* = 372	*n* = 21	value	*n* = 18	*n* = 18	value	
**Preoperative characteristics**								
Age, (years)		53.24 ± 11.33	56.10 ± 10.32	0.260	53.56 ± 10.43	54.83 ± 10.29	0.714	0.123
Gender				0.040			1.000	0.000
Male, n, (%)		282(75.8)	20(95.2)		17(94.4)	17(94.4)		
Female, n, (%)		90(24.2)	1(4.8)		1(5.6)	1(5.6)		
BMI		26.78 [25.25,28.30]	27.20 [25.25,29.15]	0.034	28.01 ± 3.47	27.73 ± 4.10	0.831	0.074
Smoke, n, (%)		149(40.0)	11(52.4)	0.263	10(55.6)	10(55.6)	1.000	0.000
Drink, n, (%)		92(24.7)	6(28.6)	0.692	6(33.3)	6(33.3)	1.000	0.000
**History of diseases**								
Structural Lung Disease		15(4.0)	1(4.8)	0.869	1(5.6)	1(5.6)	1.000	0.000
Stroke		21(5.6)	4(19.0)	0.014	1(5.6)	1(5.6)	1.000	0.000
Cardiac Surgery		4(1.1)	0(0.0)	0.633	0(0.0)	0(0.0)		

BMI, body mass index; PSM, propensity score matching

## Reasons for extubation failure

Among the 393 patients included in the study, 21 (5.3%) experienced extubation failure. Among these, the median time to reintubation was 96 h (IQR: 24–144 h). Early reintubation (≤ 24 h) occurred in 5 patients (23.8%), while late reintubation (> 24 h) occurred in 16 patients (76.2%). All causes of extubation failure in the 21 patients (including the 3 patients ultimately excluded from paired analysis) are listed. Approximately half of the patients experienced extubation failure due to respiratory-related factors, including respiratory failure caused by pulmonary infections and excessive airway secretions. The remaining patients primarily failed extubation due to newly occurring postoperative arrhythmias, newly developed neurological disorders, or surgery-related complications such as bleeding, cardiac tamponade, and surgical site infections (Table 2).

**Table 2:** Reasons of extubation failure

Category and specific cause		*n* = 21(%)	Early (≤ 24 h, *n* = 5)	Late (> 24 h, *n* = 16)
**ATAAD-specific complications**		**4 (19.0)**	**0**	**4**
	Stroke or Paraplegia	3 (14.3)	0	3
	Recurrent Laryngeal Nerve Injury	1 (4.8)	0	1
**Post-cardiac surgery complications**		**5 (23.8)**	**3**	**2**
	Hemorrhagic	2 (9.5)	2	0
	Thoracic incision infection	2 (9.5)	0	2
	Pericardial Tamponade	1 (4.8)	1	0
**General ICU-related complications**		**12 (57.1)**	**2**	**10**
	Pulmonary Infection or Secretions	10 (47.6)	1	9
	Obstructive Sleep Apnea	1 (4.8)	0	1
	Cardiac arrest	1 (4.8)	1	0

### Perioperative characteristics and outcomes of patients after PSM

After PSM, there were statistically significant differences in the preoperative APACHE Ⅱ score, GERAADA score and hospital LOS between the two groups. Patients with extubation failure had higher preoperative APACHE Ⅱscores (10 ± 1.94 vs. 12.89 ± 3.07, *P* = 0.002), GERAADA scores (19.06 ± 10.81 vs. 25.14 ± 6.25, *P* = 0.046) and LOSs (17.94 ± 5.94 vs. 29.22 ± 17.00, *P* = 0.015). Although the extubation failure group had higher rates of in-hospital mortality (0% vs. 11.1%), 90-day mortality (5.6% vs. 22.2%) and longer ICU stay (5.56 [3.58,7.53] vs. 11.28 [6.77,15.78]), these differences were not statistically significant. As a retrospective study without subjective intervention, the use of non-invasive ventilation (NIV), high-flow nasal cannula (HNFC), or alveolar recruitment therapy was determined by the attending physicians, with no significant difference in the number of patients receiving these treatments between the two groups. Furthermore, there were no significant differences in preoperative oxygenation, extubation-phase oxygenation, carbon dioxide retention, operation time, CPB time or ACC time (Table 3).

**Table 3:** Characteristics of patients between two groups

-		Extubation group	Failure group		*P*
Variables		*n* = 18	*n* = 18		value
**Preoperative characteristics**					
	APACHE Ⅱ score	10.00 ± 1.94	12.89 ± 3.07		0.002
	GERAADA, %	19.06 ± 10.81	25.14 ± 6.25		0.046
	P/F	261.73 ± 88.44	272.63 ± 106.52		0.741
**Intraoperative Characteristics**					
	OP time, h	7.24 ± 1.39	6.92 ± 1.34		0.493
	CPB time, min	186.61 ± 55.40	172.06 ± 35.19		0.353
	ACC time, min	107.89 ± 35.48	100.39 ± 29.49		0.495
**Peri-extubation Characteristics**					
	APACHE Ⅱ score	15.61 ± 2.64	15.83 ± 4.02		0.846
	EuroSCORE	8.17[7.36,8.97]	7.67[7.08,8.26]		0.389
	P/F before extubation	180.39 ± 93.65	222.55 ± 110.85		0.226
	P/F during SBT	227.37 ± 45.70	265.96 ± 83.46		0.097
	P/F 2 h after extubation	221.66 ± 69.48	197.57 ± 69.99		0.307
	Pco_2_ during SBT, mmHg	33.42 ± 4.04	33.93 ± 4.09		0.709
	Pco_2_ 2 h after extubation, mmHg	35.93 ± 5.98	37.03 ± 5.47		0.570
	MV time, h	57.23 ± 36.84	60.35 ± 52.62		0.838
	NIV, n (%)	3(16.7)	4(22.2)		0.500
	HFNC, n (%)	3(16.7)	6(33.3)		0.222
	Alveolar Recruitment, n (%)	6(33.3)	4(22.2)		0.356
**Complication**					
	Stroke, n (%)	1 (5.6)	5 (27.8)		0.177
	Paraplegia, n (%)	3 (16.7)	3 (16.7)		1.000
**Outcomes**					
	ICU LOS, d	5.56[3.58,7.53]	11.28[6.77,15.78]		0.143
	LOS, d	17.94 ± 5.94	29.22 ± 17.00		0.015
	In hospital mortality, n (%)	0	2(11.1)		0.243
	90-day Mortality, n (%)	1(5.6)	4(22.2)		0.169

OP, operation; CPB, cardiopulmonary bypass; ACC, aortic cross clamp; P/F, PaO_2_/FIO_2_; SBT, spontaneous breathing trial; MV, mechanical ventilation; NIV, non-invasive ventilator; HFNC, high-flow nasal cannula oxygen therapy; LOS, hospital length of stay

### Reasons for mechanical ventilation duration greater than 48 h

Among the 36 patients, the earliest extubation occurred at 8 h post-operatively, whereas the latest extubation took place at 161.7 h. A total of 17 patients, accounting for approximately half of the cohort, required mechanical ventilation for more than 48 h. Given that the mean mechanical ventilation (MV) time was more than 48 h in both groups in this study, we collated the reasons for delayed extubation. Four of them experienced delayed extubation due to delayed recovery from anaesthesia, 1 was unable to cooperate during the spontaneous breathing trial (SBT) due to delirium, and the other 6 experienced delayed extubation because of pulmonary infections and acute respiratory distress syndrome (ARDS). Additionally, 4 patients experienced delayed extubation due to stroke or paraplegia. Among these patients, some were successfully extubated after partial recovery of neurological function, whereas others discontinued mechanical ventilation following tracheostomy (Table 4).

**Table 4:** Reasons of MV duration over 48 h

Variables		*n* = 17	%
Delayed recovery from anaesthesia		4	11.1
Delirium		1	2.8
Stroke or Paraplegia		4	11.1
SBT failure		1	2.8
ARDS		3	8.3
Pulmonary Infection		3	8.3
Unstable Circulation		1	2.8

ARDS, Acute Respiratory Distress Syndrome

### Sensitivity and specificity of the APACHE II score and GERAADA in predicting extubation failure

**Fig. 2:**
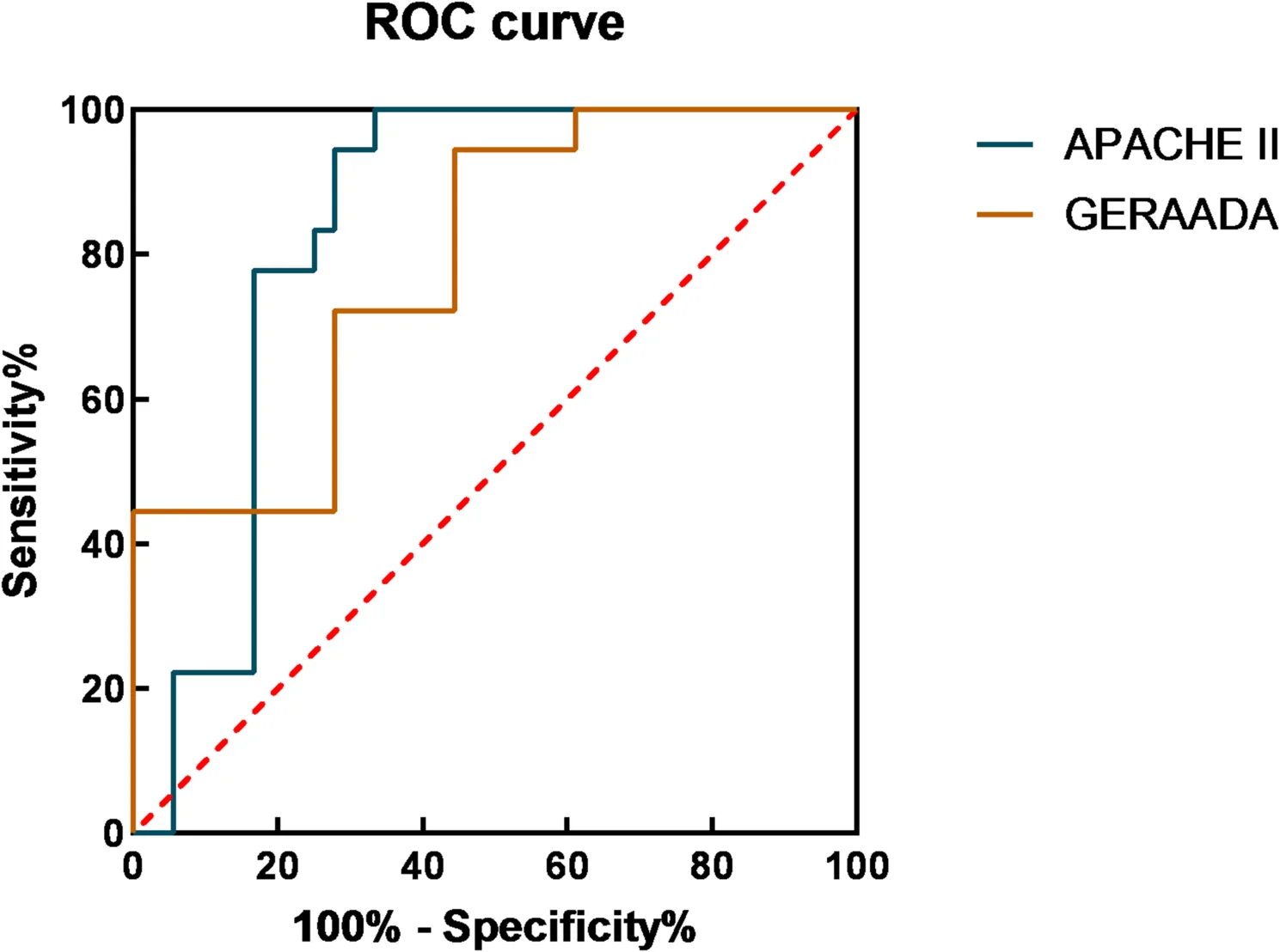
ROC curves for the APACHE Ⅱ score and GERAADA in predicting extubation failure

Preoperative APACHE Ⅱ and GERAADA scores were significantly different between patients with successful extubation and those with failed extubation. Therefore, receiver operating characteristic (ROC) curve analysis was employed to compare the sensitivity and specificity of both scores in predicting extubation risk. The analysis revealed that GERAADA had a cut-off value of 21.9%, with an AUC of 0.832 (95% CI: 0.680–0.983), outperforming the APACHE Ⅱ cut-off value of 12.5 (AUC = 0.744, 95% CI: 0.584–0.904). DeLong test showed no statistically significant difference between the two AUCs (*p* = 0.485). (Fig. 2).

### Univariate and multivariate associations with the APACHE II score and GERAADA score

To further elucidate the association between preoperative APACHE Ⅱ score, GERAADA score, and extubation failure, univariate analyses were performed incorporating: (1)preoperative parameters (demographic characteristics and PaO₂/FiO₂ ratio), (2) intraoperative variables (cardiopulmonary bypass time, operative time, and aortic cross-clamp time), and (3) pre-extubation indicators (APACHE Ⅱ score after ICU admission, PaO₂/FiO₂ ratio at ICU admission, pre-extubation PaO₂/FiO₂ ratio, and pre-extubation PCO₂).

The results demonstrated that in univariate analysis, elevated preoperative APACHE Ⅱ score was strongly associated with an increased risk of extubation failure (OR = 1.63, *P* = 0.012), while the GERAADA score showed a trend toward association (OR = 1.11, *P* = 0.071).

Subsequently, in the multivariable model incorporating both preoperative APACHE Ⅱ and GERAADA scores, preoperative APACHE Ⅱ remained independently associated with extubation failure (adjusted OR = 1.66, *P* = 0.023). Variance inflation factors (VIF = 1.009 for both predictors) confirmed the absence of multicollinearity (Table 5).

**Table 5:** Univariate and Multivariate Analysis of Risk Factors

Variables		Univariate		Multivariate	*P* Value	VIF
		OR (95% CI)	*P* Value	Adjusted OR (95% CI)		
**Preoperative characteristics**						
	APACHE Ⅱ score	1.63 (1.11–2.39)	0.012	1.66 (1.07–2.57)	0.023	1.009
	GERAADA, %	1.11 (0.99–1.25)	0.071	1.09 (0.97–1.22)	0.157	
	P/F	1.00 (0.99–1.01)	0.733			
**Intraoperative Characteristics**						
	OP time	0.84 (0.51–1.38)	0.481			
	CPB time	0.99 (0.98–1.01)	0.347			
	ACC time	0.99 (0.97–1.01)	0.485			
**Pre-extubation Characteristics**						
	APACHE Ⅱ score	1.02 (0.84–1.24)	0.840			
	EuroSCORE	0.77 (0.47–1.26)	0.294			
	P/F before extubation	1.00 (1.00-1.01)	0.224			
	P/F during SBT	1.01 (1.00-1.02)	0.102			
	Pco_2_ during SBT	1.03 (0.88–1.22)	0.700			
	MV time	1.00 (0.99–1.02)	0.833			
	Alveolar Recruitment	0.57 (0.13–2.51)	0.459			

BMI, body mass index; PSM, propensity score matching; OP, operation; CPB, cardiopulmonary bypass; ACC, aortic cross clamp; P/F, PaO2/FIO2; SBT, spontaneous breathing trial; MV, mechanical ventilation; VIF, variance inflation factors

## Discussion

Our findings suggest that among patients undergoing surgery for aortic dissection, when baseline characteristics are comparable through propensity score matching, neither operative duration, intraoperative events, nor post-extubation management strategies (NIV/HFNC) demonstrate a significant association with extubation failure. Only preoperative APACHE II and GERAADA scores remained significantly different between the two matched groups. The robust performance of preoperative APACHE Ⅱ score across both univariate (OR = 1.63, *P* = 0.012) and multivariable regression analyses (adjusted OR = 1.66, *P* = 0.023).

The extubation failure rate in our cohort was 5.3% (21/393), consistent with published rates ranging from 4.4% to 16.4% [9–11]. Among all patients, the mean ventilation duration was 55.7 h, with 42.5% requiring ventilation beyond 48 h. In the matched cohort (*n* = 36), the mean ventilation duration was 58.8 h, and 47.2% exceeded 48 h, indicating that prolonged ventilation was not attributable to selection bias. The US Society of Thoracic Surgeons (STS) has proposed defining delayed extubation as the failure to extubate a cardiac surgical patient 24 h after leaving the operating theatre [12]. Extubation beyond 48 h has also been used as a cut-off for delayed extubation in some studies [2]. However, there are no exact criteria for defining delayed extubation time after aortic dissection. We reviewed some recent Chinese studies on aortic dissection and reported that the average duration of postoperative ventilation for patients ranged from 19 to 62 h [13–15].

In this study, the strictest criteria were applied to define extubation failure: any occurrence of reintubation or emergency tracheostomy following extubation during the hospitalization period was classified as extubation failure. Notably, among the 21 patients who failed extubation, 5 (57%) experienced early failure (≤ 24 h), predominantly due to surgical bleeding (hemorrhagic, Pericardial tamponade, *n* = 3), while 16 (43%) had delayed failure (> 24 h), primarily from pneumonia (*n* = 9) and the inability to cough up phlegm caused by neurological complications (paraplegia and cerebral infarction, *n* = 5), which eventually led to respiratory failure. This temporal distinction suggests that early failure was more frequently attributable to immediate postoperative surgical complications, whereas delayed failure predominantly arose from compromised airway clearance mechanisms. Although the prevalence of stroke and paraplegia did not differ significantly between the extubation success and failure groups in our matched cohort. Patients with neurological deficits remain at high risk for extubation failure due to impaired cough and airway self-cleaning capacity. The prophylactic use of NIV or HFNC after extubation have been reported to reduce the risk of extubation failure in some study [5–6, 16]. Our study also found no significant difference in the use of NIV or HFNC between the success and failure groups after propensity score matching. Given the established importance of maintaining adequate oxygenation and the widespread use of prophylactic oxygen therapy, optimizing airway clearance has emerged as a critical priority for improving extubation success rates. Our center has therefore introduced non-invasive mechanical insufflation-exsufflation devices to enhance secretion clearance in high-risk patients.

Patients who were successfully extubated had lower preoperative APACHE II scores than those who experienced extubation failure, whereas postoperative APACHE II scores were similar between the two groups and generally higher than preoperative values. This convergence postoperative likely reflects the universal physiological stress of major surgery. Importantly, preoperative APACHE II score maintained strong associations in both univariate (OR = 1.63, *P* = 0.012) and multivariate regression (adjusted OR = 1.66, *P* = 0.023), confirming its independent predictive capacity. Most existing studies do not specify whether APACHE II was assessed preoperatively or postoperatively [2, 6], which may reflect differences in admission pathways. Our findings suggest that the preoperative assessment carries prognostic value for extubation outcomes.

The GERAADA score is a risk prediction tool recently developed specifically for aortic dissection patients in Europe [17]. It is generally used to predict early postoperative mortality in dissection patients by scholars from various countries [18–21]. The GERAADA score also differed significantly between groups. However, in multivariable analysis, GERAADA did not retain statistical significance (OR = 1.09, *P* = 0.157). ROC analysis demonstrated acceptable discriminative ability and a meaningful predictive value for both scores (APACHE II AUC = 0.744; GERAADA AUC = 0.832), and the DeLong test indicated no statistically significant difference between the two AUCs (*P* = 0.485). Given the relative accessibility and consistent performance of APACHE II, it may be more practical for preoperative risk stratification in many clinical settings.

Based on these exploratory findings, our center now routinely obtains preoperative APACHE II scores for all acute aortic dissection patients without immediate life-threatening emergencies. Patients with scores exceeding 12.5 receive intensified monitoring and a proactive management bundle: more active lung-protective strategies during mechanical ventilation, prophylactic post-extubation support with NIV or HFNC, postoperative respiratory rehabilitation (emphasizing deep breathing, effective coughing, expectoration, and assisted coughing techniques), and targeted delirium prevention based on our center’s prediction model [22]. Additionally, for patients receiving post-extubation respiratory support, we incorporate the VOX and ROX indices to assist in reintubation risk assessment [23, 24].

In this study, all patients included in the analysis completed a 3-year follow-up. With the exception of one patient in the extubation failure group who died of recurrent cerebral hemorrhage one year after discharge, all other patients survived. Among them, one patient with paraplegia recovered lower limb muscle strength and was able to stand with assistance.

We are aware of the shortcomings of our study. First, as a retrospective observational study, we could not collect all potentially relevant data and details, such as preadmission infections and pathogenicity tests, perioperative point-of-care ultrasound results, and invasive hemodynamic data. Second, operative complexity is partially reflected by the GERAADA score, and hemodynamic status is captured by the APACHE II score. Nevertheless, residual confounding from unmeasured preoperative factors not captured by these scores (e.g., frailty, detailed intraoperative events) cannot be completely excluded. Third, the small number of events (*n* = 21) limits statistical power, and the derived cutoffs (APACHE II > 12.5; GERAADA > 21.9%) are exploratory, requiring prospective validation. This was a single-centre study, therefore, the results might have several limitations, and we look forward to the subsequent refinement of further studies.

## Conclusion

APACHE II score greater than 12.5 or a GERAADA score exceeding 21.9% may indicate an increased risk of extubation failure, warranting close postoperative monitoring and careful extubation evaluation. Preoperative APACHE II score demonstrated robust predictive performance in both univariate (OR = 1.63, *P* = 0.012) and multivariable regression analyses (adjusted OR = 1.66, *P* = 0.023). Given that delayed failure was primarily driven by impaired airway clearance, post-extubation management should prioritize airway clearance strategies alongside conventional oxygen support. These exploratory findings require prospective validation.

## Data Availability

The datasets used and analyzed during the current study are available from the corresponding author on reasonable request.

## Abbreviations

ATAAD: Acute Stanford type A aortic dissection
ACC: Aortic cross-clamp
APACHE Ⅱ: Acute Physiology and Chronic Health Evaluation Ⅱ
ARDS: Acute respiratory distress syndrome
BMI: Body mass index
CI: Confidence intervals
CPB: Cardiopulmonary bypass
ERAS: Enhanced Recovery After Surgery
EuroScore: European System for Cardiac Operative Risk Evaluation
FiO_2_: Fractional inspired oxygen
FTCA: Fast Track Cardiac Anaesthesia
GERAADA: German Registry for Acute Aortic Dissection Type A
HFNC: High-flow nasal cannula
ICU: Intensive care unit
IQ: Rinter quartile range
LOS: Length of stay
MV: Mechanical ventilation
NIV: Noninvasive ventilation
OR: Odds ratios
PaCO_2_: Partial pressure of arterial carbon dioxide
PSM: Propensity score matching
PSV: Pressure Support Ventilation
ROC: Receiver operating characteristic
ROX: Rate-Oxygenation
SBT: Spontaneous breathing trial
SD: Standard deviation
VIT: Variance inflation factors
VOX: Volume-Oxygenation
